# *Grifola frondosa* Extract Containing Bioactive Components Blocks Skin Fibroblastic Inflammation and Cytotoxicity Caused by Endocrine Disrupting Chemical, Bisphenol A

**DOI:** 10.3390/nu14183812

**Published:** 2022-09-15

**Authors:** Ju-Ha Kim, Seong-Ryeong Lim, Dae-Hwa Jung, Eun-Ju Kim, Junghee Sung, Sang Chan Kim, Chang-Hyung Choi, Ji-Woong Kang, Sei-Jung Lee

**Affiliations:** 1 Department of Public Health, Daegu Haany University, Gyeongsan 38610, Korea; 2 Department of Pharmaceutical Engineering, Daegu Haany University, Gyeongsan 38610, Korea; 3 RFBio Research & Development Center, RFBio Co., Ltd., Gunpo-si 15807, Korea; 4 College of Korean Medicine, Daegu Haany University, Gyeongsan 38610, Korea; 5 Division of Cosmetic Science and Technology, Daegu Haany University, Gyeongsan 38610, Korea

**Keywords:** apoptotic cell death, bisphenol A, *Grifola frondosa*, normal human dermal fibroblasts, pyroptosis, reactive oxygen species

## Abstract

*Grifola frondosa* (GF), a species of *Basidiomycotina*, is widely distributed across Asia and has been used as an immunomodulatory, anti-bacterial, and anti-cancer agent. In the present study, the pharmacological activity of the GF extract against an ecotoxicological industrial chemical, bisphenol A (BPA) in normal human dermal fibroblasts (NHDFs), was investigated. GF extract containing naringin, hesperidin, chlorogenic acid, and kaempferol showed an inhibitory effect on cell death and inflammation induced by BPA in the NHDFs. For the cell death caused by BPA, GF extract inhibited the production of reactive oxygen species responsible for the unique activation of the extracellular signal-regulated kinase. In addition, GF extract attenuated the expression of apoptosis-related proteins (Bax, Bcl-2, and cleaved caspase-3) and the pro-inflammatory cytokine IL-1β by the suppression of the redox-sensitive transcription factor, nuclear factor-kappa B (NF-κB) in BPA-treated NHDFs. For the inflammation triggered by BPA, GF extract blocked the inflammasome-mediated caspase-1 activation that leads to the secretion of IL-1β protein. These results indicate that the GF extract is a functional antioxidant that prevents skin fibroblastic pyroptosis induced by BPA.

## 1. Introduction

A well-known endocrine disruptor, Bisphenol A (BPA) is widely used to produce polycarbonate plastics and epoxy resins, and is suspected to cause problems related to reproductive development and function [1,2,3,4]. While contaminating food and drink through their packaging materials was believed to be the major exposure route of BPA [5], the toxicological role of BPA through dermal contact and absorption in the skin has not been well investigated. The skin plays a critical role in protecting the body from a variety of environmental insults and consists of two main structural layers: the epidermis and the dermis [6]. Unlike the epidermis, which mainly consists of keratinocytes [7], the dermis is largely composed of a dense, collagen-rich extracellular matrix (ECM) that provides structural and mechanical support for the skin [8]. Given the fact that BPA exposure routes through dermal contact and absorption could not be ignored [9], many reports have indicated that BPA induces skin dermal damage and thus causes the production of pro-inflammatory proteins associated with human skin diseases [10,11].

In several experimental models, the fundamental mechanism of toxicity induced by BPA is known to be associated with oxidative stress and inflammation responsible for important pathophysiological processes, promoting lipid peroxidation and cell death due to the overexpression of certain genes and proteins [12,13,14,15]. While apoptosis is related to caspase-3-dependent cell death associated with DNA fragmentation and cell shrinkage, pyroptosis has been known as an inflammatory cell death accompanied by inflammasome-mediated caspase-1 activation that results in maturation of the pro-inflammatory cytokines IL-1β and IL-18 [16]. Indeed, recent studies have reported that apoptotic inflammatory damage is considered as a main feature of dermatitis in skin pathophysiology including abnormal telangiectasia, inflammation, increased wrinkles, and pigmentation [9,17]. Nuclear factor-kappa B (NF-κB) is a direct transcriptional target for expression of numerous pro-inflammatory cytokines (e.g., IL-1β, IL-6, TNF-α) and apoptotic genes (e.g., Bax, Bcl-2) to propagate and maintain the skin’s inflammatory responses [18,19,20]. Given that BPA induces NF-κB activation and evokes the expression of apoptotic signaling proteins and proinflammatory cytokines associated with various manifestations of skin damage, it is important to find pharmacological substances that ameliorate the risk of many types of inflammatory skin damage caused by BPA exposure.

Many medicinal mushrooms and their bioactive components have been reported to have a wide range of skin preventive properties and pharmacological functions against the apoptotic pathway mediated by ROS in skin cells [21,22]. Among these, medicinal mushrooms, *Ganoderma, Tramete*, *Flammulina*, *Lentinus*, *Auricularia*, and *Grifola* have shown profound antioxidative and medical properties [23,24]. *Grifola frondosa* (GF), also known as maitake, is considered an edible healthy mushroom with a sweet and special taste due to its high content of trehalose, 5′-nucleotide, and aspartic and glutamic acid [25]. Many bioactive components of GF have been found including carbohydrates, minerals, proteins, vitamins, and dietary fibers. In addition to its nutritional value, many reports have suggested that GF possesses various biological and pharmacological effects such as antitumor, anti-inflammatory, antidiabetic, and antioxidant activities [26,27]. GF has also received attention due to its physiological roles in the suppression of Enterovirus activity and the alleviating of hepatic steatosis and liver inflammation [28,29]. However, the functional role of GF in skin fibroblastic inflammation and cytotoxicity caused by BPA exposure has not yet been explored. Therefore, in this study, the functional role of GF during oxidative apoptotic cell death and inflammation induced by BPA in human dermal fibroblasts was investigated to define the cellular signaling cascade underlying the nutraceutical effects of GF with regard to skin dermal protection.

## 2. Materials and Methods

### 2.1. Materials

Bisphenol A (BPA), methanol (purity ≥99.9%), ethanol (purity ≥99.9%), acetonitrile (purity ≥99.9%), formic acid (FA, purity ≥97.5%), Whatman^®^ filter paper, skim milk powder, chlorogenic acid (purity ≥95.0%), caffeic acid (purity ≥99.0%), p-coumaric acid (purity ≥98.0%), naringin (purity ≥95.0%), hesperidin (purity ≥97.0%), and kaempferol (purity ≥97.0%) were obtained from Sigma-Aldrich (St. Louis, MO, USA). β-actin, Bcl-2, Bax, cleaved caspase-3, ASC, caspase-1, NF-κBp65, IκBα, JNK, p38 MAPK, and ERK antibodies were obtained from Santa Cruz Biotechnology (Paso Robles, CA, USA). HRP goat anti-mouse/rabbit antibodies were obtained from Abcam (Cambridge, MA, USA). Bay 11-7082, PD98059, and N-acetylcysteine (NAC) were purchased from Tocris (Minneapolis, MN, USA).

### 2.2. Preparation of Grifola frondosa Extraction

The fruiting bodies from *Grifola frondosa* (GF) were purchased in November 2020 from the Ipsae Mushroom Gwa Frields Co., Ltd. (Gyeongsangnam-do, Jinju, Korea). The dried GF (3.0 g) were chopped and soaked in ddH_2_O (300 mL) at 25 °C for 72 h. The GF solution was filtered by using Whatman^®^ filter paper (No. 2) to remove debris, and then dried with a freeze-dryer (FDB-5503; Operon, Gyeonggi-do, Korea). The resulting crude extracts of GF were stored at −20 °C to prevent the growth of microorganisms. The final production of GF crude extracts yielded about 0.22 g (7.3%) from the raw material. For the experiments, cells were exposed to the GF crude extracts dissolved in ddH_2_O at 25 °C.

### 2.3. Ultra-Performance Liquid Chromatography (UPLC)

UPLC was conducted in Acquity UPLC system equipped with a binary solvent manager pump, an optical detector (photodiode array, and a column heater (Waters, Prague, Czech Republic). Chromatographic separation was done using a BEH column (2.1 mm × 100 mm i.d., 1.7 µm). For quantitative data analysis, the flavonol and polyphenolic compounds from the crude extracts of GF (0.5 g) were extracted by using an ultrasonic microwave extractor Powersonic 505; Hwashin Tech, Daegu, South Korea) in 70% methanol (10 mL) for 1 h. The amounts of UPLC standards (chlorogenic acid, caffeic acid, p-coumaric acid, naringin, kaempferol, and hesperidin) were dissolved in methanol to get the stock solutions to 1 mg/mL. To obtain the working solutions, each standard solution was diluted in methanol at concentrations of 12.5, 25, 50, and 100 µg/mL. The analysis conditions are shown in Table 1. The coefficient (R^2^) value of all standard materials exceeded 0.999. The flow rate and the injection volume were 0.4 mL/min and 2 µL, respectively. The mobile phase consisted of a 16 min gradient system combining water and acetonitrile containing 0.1% aqueous formic acid (FA). Data were confirmed by using Empower 3 software (Waters^®^, Prague, Czech Republic). The chromatograms were recorded at a wavelength of 254 nm for caffeic acid and kaempferol, 280 nm for naringin and hesperidin, 285 nm for chlorogenic acid, and 310 nm for p-coumaric acid. Peaks were identified by confirming retention times and were quantitated by internal standards.

### 2.4. Cells

Normal human dermal fibroblasts were obtained from the American Type Culture Collection (ATCC, Manassas, VA, USA). Cells were cultured in an incubator maintained at 36.5 °C with 5% CO_2_ and grown in RPMI-1640 containing penicillin (100 U/mL), streptomycin (100 μg/mL), and 10% FBS, respectively.

### 2.5. Measurement of Total Polyphenols Content

Total polyphenolic content was determined by a total phenols colorimetric assay kit (Elabscience, Houston, TX, USA) according to the manufacturer’s instructions. GF crude extract (0.1 g/mL) dissolved in 60% ethanol was sonicated with a frequency of 60 kHz and then collected the supernatant by centrifugation at 12,000× *g* for 8 min. The resulting supernatant mixed with the reagent consisting of the Folin-phenol and the alkali was analyzed by using the 96 well microplate reader (SPARK, Seestrasse, Männedorf, Switzerland) at 760 nm. The total phenolic content of the GF crude extract was determined using a standard curve of O-dihydroxybenzene.

### 2.6. Measurement of Total Flavonoids Content

Total flavonoid content was determined by a total flavonoid colorimetric assay kit (Elabscience, Houston, TX, USA) according to the manufacturer’s instructions. GF crude extract (0.02 g/mL) dissolved in 60% ethanol was sonicated with a frequency of 60 kHz and then collected the supernatant by centrifugation at 2000× *g* for 8 min. A supernatant mixed with the reagent consisting of NaCl, aluminum, and alkali was analyzed by using the 96 well microplate reader (SPARK, Seestrasse, Männedorf, Switzerland) at 510 nm. The total flavonoids content of GF crude extract was determined using a standard curve of quercetin.

### 2.7. Cell Proliferation Assay

An EZ-CYTOX kit (Dail-Lab Service, Seoul, Korea) was used to determine cell proliferation of NHDFs treated with BPA and GF according to the manufacturer’s instructions. Cells were incubated with 10 μL of EZ-CYTOX master mix for 1 h. Cell proliferation was directly analyzed by measuring the absorbance at 450 nm. The absorbance was detected using a microplate reader (SPARK, Seestrasse, Männedorf, Switzerland).

### 2.8. Intracellular Reactive Oxygen Species (ROS) Detection

To quantify the intracellular ROS levels, NHDFs treated with 10 μM of 2′,7′-dichlorofluorescein diacetate (CM-H_2_DCFDA) for 30 min were washed twice with PBS. The cell suspension (100 μL) was loaded into a 96 well plate and the level of ROS was measured by using a fluorescent microplate reader (SPARK, Seestrasse, Männedorf, Switzerland) at the wavelengths of 485 nm (excitation) and 535 nm (emission), respectively.

### 2.9. Western Blot Analysis

Western blot analysis for detecting protein expression was conducted as previously described [30]. The intensity of protein band confirmed by the chemiluminescence was quantified by using Scion Image software (Scion Image Beta 4.02, Frederick, MD, USA). The relative optical density (ROD) of the protein bands was obtained after normalization of the intensities to those of β-actin.

### 2.10. Flow Cytometry

NHDFs were arrested in the G0-G1 cell cycle phase by culture in serum-free medium for 24 h before treatment with GF and BPA. Apoptotic cell death was detected with an Annexin V labeled with fluorescein isothiocyanate (FITC) (Invitrogen, Carlsbad, CA, USA) according to the manufacturer’s instructions. The cells (2 × 10^5^) were treated with Annexin V-FITC conjugate (30 μg/mL) and propidium iodide (PI, 120 ng/mL) for 15 min. The proportion of apoptotic cells was analyzed using a NucleoCounter^®^ image cytometer (ChemoMetec, Gydevang, Allerod, Denmark). Samples were gated to exclude debris (FSC area versus SSA area), and cell doublets were also excluded by measuring the FSC area versus FSC width. Samples were analyzed by using NucleoView NC-3000 software (ChemoMetec, Gydevang, Allerod, Denmark).

### 2.11. Determination of Adenosine Triphosphate (ATP) Level

The level of ATP was measured by ATP kit (Molecular Probes, Eugene, OR, USA) according to the manufacturer’s instructions. NHDFs were incubated with GF and BPA for 6 h. The cells were then resuspended in reaction buffer containing 0.5 mM D-luciferin, 12.5 μg/mL firefly luciferase, and 1 mM dithiothreitol. After a 15 min incubation, the level of cellular ATP was measured by using a fluorescent microplate reader (SPARK, Seestrasse, Männedorf, Switzerland) at excitation and emission wavelengths of 560 and 595 nm, respectively.

### 2.12. Real-Time PCR

Cellular RNA was isolated using the NucleoSpin^®^ RNA plus kit (Macherey-Nagel, Düren, Germany). To prepare the cDNA, reverse transcription was performed using a ReverTra Ace^®^ qPCR RT Master Mix (cDNA kit) (TOYOBO, Osaka, Japan). AccuPower^®^ 2X Greenstar qPCR Master Mix (Bioneer, Daejeon, Korea) was used to amplify the pro-inflammatory cytokines (IL-1β, TNF-α, and IL-6). The primer sequences used are shown in Table 2.

### 2.13. Enzyme-Linked Immunosorbent Assay (ELISA)

NHDFs were incubated with 100 µg/mL of GF and inhibitors for 30 min prior to BPA (50 μM) treatment for 6 h. The medium from the cultured NHDF was centrifuged at 5000× *g* for 15 min to remove debris at 4 °C. The level of IL-1β production was measured by using an IL-1β ELISA kit (Abcam, Cambridge, UK) following the manufacturers’ instructions.

### 2.14. Immunofluorescence

NHDF on slides was fixed in PBS-based paraformaldehyde (4%) for 15 min at 25 °C, incubated in Triton X-100 (0.1%) for 10 min, and treated with the blocking solution (normal goat serum) for 1 h at 25 °C. Samples were then stained with primary anti-rabbit ASC antibody overnight and incubated with Alexa 555-conjugated phalloidin (Thermo Fisher Scientific, Hudson, NH, USA) for 2 h. The ASC speck formation was visualized with an Olympus Fluo View™ 300 confocal microscope with a 400 × objective lens.

### 2.15. Statistical Analysis

Experiments were performed in triplicate and repeated three times. Results were represented as mean ± standard errors (S.E.). All analysis was determined by ANOVA in SPSS 16 software (IBM Corp, Armonk, NY, USA). Differences were considered statistically significant at *p* < 0.05.

## 3. Results

### 3.1. Inhibitory Effect of Grifola frondosa (GF) on Skin Cytotoxicity and Inflammation Stimulated by Bisphenol A (BPA)

**Figure 1:**
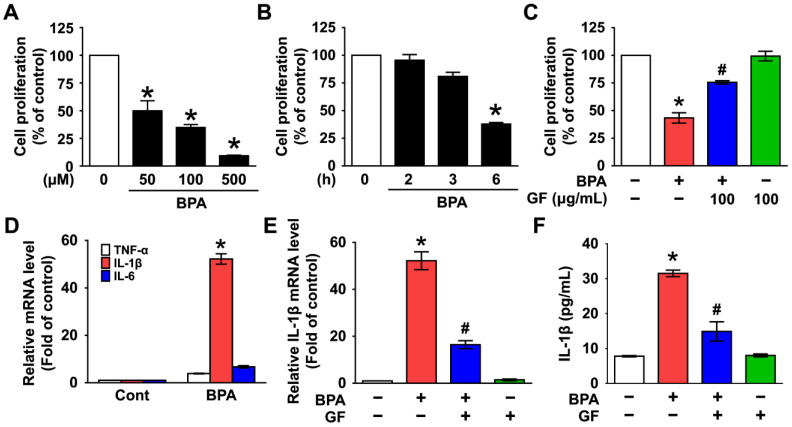
Inhibitory effect of *Grifola frondosa* (GF) on skin cytotoxicity and inflammation stimulated by bisphenol A (BPA). (**A**) Dose-dependent response of cell viability in normal human dermal fibroblasts (NHDFs) treated with BPA (0–500 μM) are shown. The cell viability was determined by EZ-CYTOX assay. n = 3. * *p* ≤ 0.05 vs. 0 μM. (**B**) Time-dependent response of cell viability treated with BPA (50 μM) is shown. n = 3. * *p* ≤ 0.05 vs. 0 h. (**C**) NHDFs were co-treated with GF (100 μg/mL) and BPA for 6 h. n = 4. * *p* ≤ 0.01 vs. Cont. # *p* ≤ 0.05 vs. BPA alone. (**D**) NHDF was treated with BPA for 6 h. The effect of BPA on the expression of pro-inflammatory cytokines was determined by qRT-PCR. * *p* ≤ 0.01 vs. Cont. n = 3. (**E**) NHDF was exposed to the BPA in the presence of GF for 6 h. The IL-1β mRNA level is shown. * *p* ≤ 0.05 versus control. # *p* ≤ 0.01 vs. BPA alone. n = 3. (**F**) The level of IL-1β production regulated by GF in BPA-treated NHDF for 6 h was quantified by ELISA. * *p* ≤ 0.01 vs. Cont. # *p* ≤ 0.01 vs. BPA alone. n = 3.

NHDFs were treated with BPA at concentrations of 0–500 µM for 6 h. BPA caused cytotoxicity of NHDFs in a dose-dependent manner (Figure 1A). A decrease in cell viability resulted after treatment with 50 µM of BPA for 6 h (Figure 1B). NHDFs were co-treated with BPA and 100 µg/mL of GF. The treatment of GF effectively decreased the cytotoxicity caused by BPA (Figure 1C). On the other hand, BPA induced the expressions of interleukin (IL)-1β mRNA at 6 h, whereas for TNF-α and IL-6, a marginal effect was noted (Figure 1D). Importantly, the expression (Figure 1E) and the secretion of IL-1β (Figure 1F) induced by BPA were markedly decreased by treatment with GF. These results indicate the pharmacological potentials of GF on skin dermal fibroblastic cytotoxicity and inflammation induced by BPA.

### 3.2. GF Contains Anti-Oxidative Components That Scavenge Intracellular ROS Caused by BPA

**Figure 2:**
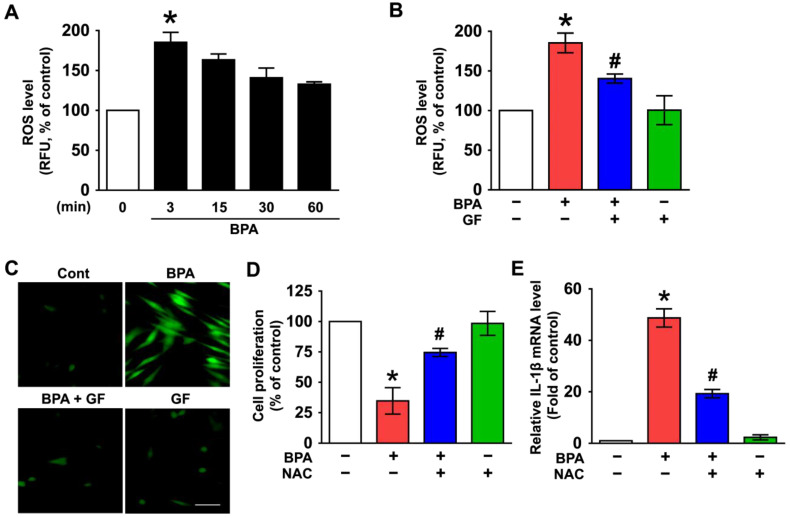
GF contains anti-oxidative components that scavenge the intracellular ROS caused by BPA. (**A**) Time-dependent responses of reactive oxygen species (ROS) production in NHDFs treated with BPA are shown. The level of ROS was determined by staining NHDFs with CM-H_2_DCFDA. n = 4. * *p* ≤ 0.05 vs. 0 min. (**B**) NHDFs were co-treated with GF and BPA for 3 min. n = 4. * *p* ≤ 0.05 vs. control. # *p* ≤ 0.01 vs. BPA alone. RFU, Relative fluorescence units. (**C**) The blocking effects of GF on ROS production (green) confirmed by confocal microscopy are shown. Scale bars, 100 μm (magnification × 100). n = 3. NHDFs were pretreated with 1 μM of N-acetylcysteine (NAC) for 30 min prior to BPA treatment for 6 h. The level of cell proliferation (**D**) and IL-1β mRNA (**E**) is shown. * *p* ≤ 0.01 versus control. # *p* ≤ 0.01 vs. BPA alone. n = 3.

BPA has been shown to evoke the production of reactive oxygen species (ROS), which amplify the signals for inflammatory skin dermal damage [31,32,33,34]. A significant augmentation in the level of intracellular ROS was observed at 3 min after incubation with BPA (Figure 2A) that was inhibited by GF treatment (Figure 2B). The ROS scavenging effect of GF was further visualized by staining NHDFs with a fluorescent CM-H_2_DCFDA (Figure 2C). To clarify the involvement of ROS in cytotoxicity and inflammation induced by BPA, NHDFs were treated with an antioxidant, N-acetylcysteine (NAC). Cytotoxicity (Figure 2D) and IL-1β mRNA expression (Figure 2E) induced by BPA were significantly inhibited by the pretreatment with NAC. These data suggest that the pharmacological effect of GF is related to its antioxidative potential against BPA.

**Figure 3:**
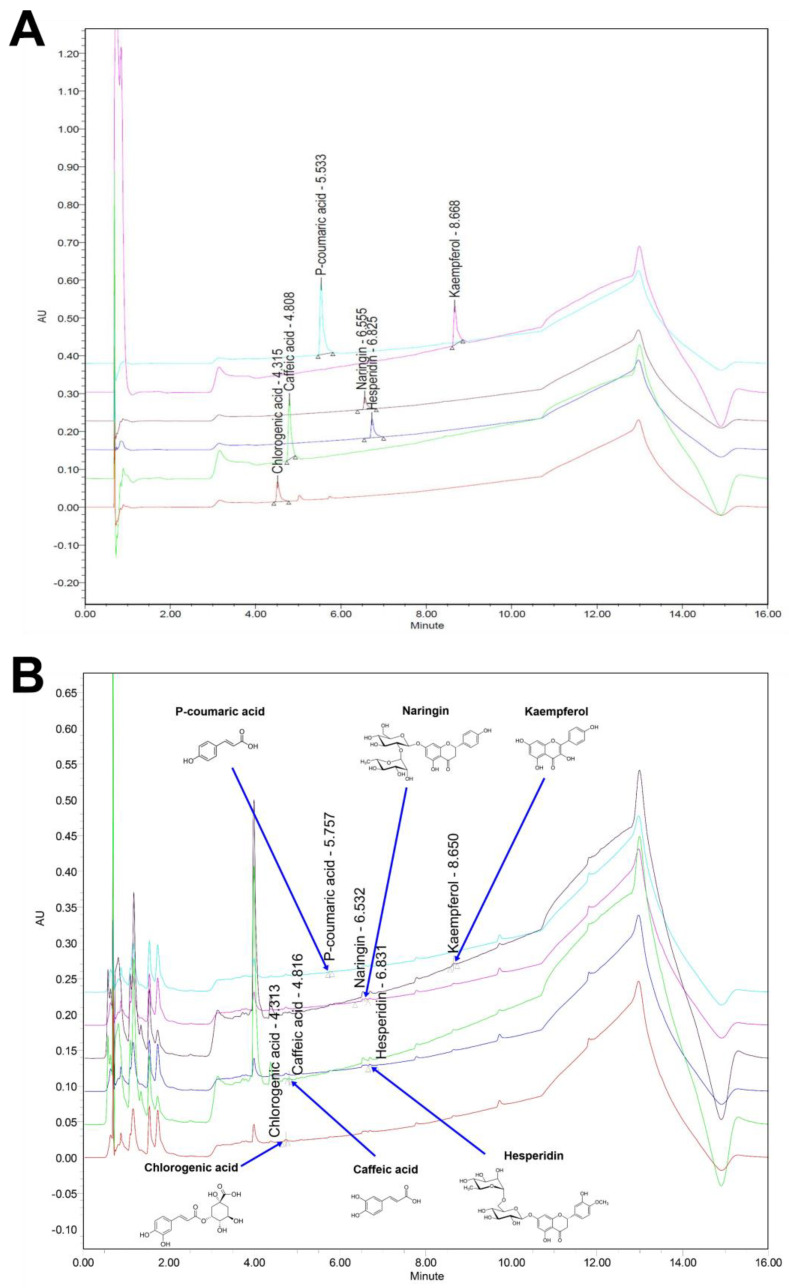
The GF contains flavonol and polyphenolic compounds responsible for its antioxidant properties. (**A**) UPLC profile of the internal standard compounds. (**B**) UPLC profile of six major compounds in GF. The insets indicate the chemical structures of naringin, hesperidin, p-coumaric acid, chlorogenic acid, kaempferol, and caffeic acid, respectively.

To find the underlying cause of pharmacological activity, the total antioxidant contents of GF extract were examined. The total polyphenolic and flavonoid contents of GF extract were 53.30 and 59.28 μg/mL, respectively (Table 3). Considering the anti-oxidative components of GF revealed by the UPLC system (Figure 3 and Table 4), the concentrations of polyphenols including caffeic acid, chlorogenic acid, and p-coumaric acid were 1.887 ± 0.148 mg/L, 4.688 ± 0.440 mg/L, and 4.794 ± 0.176 mg/L, respectively. On the other hand, the amounts of kaempferol, naringin, and hesperidin as flavonols in GF were 4.169 ± 0.227 mg/L, 18.183 ± 0.962 mg/L, and 7.488 ± 0.270 mg/L, respectively. These data suggest that GF has substantial amounts of flavonol and polyphenolic compounds responsible for their antioxidant properties against cytotoxicity and inflammation induced by BPA in human dermal fibroblasts.

### 3.3. GF Inhibits the Phosphorylation of ERK in BPA-Induced NHDFs

**Figure 4:**
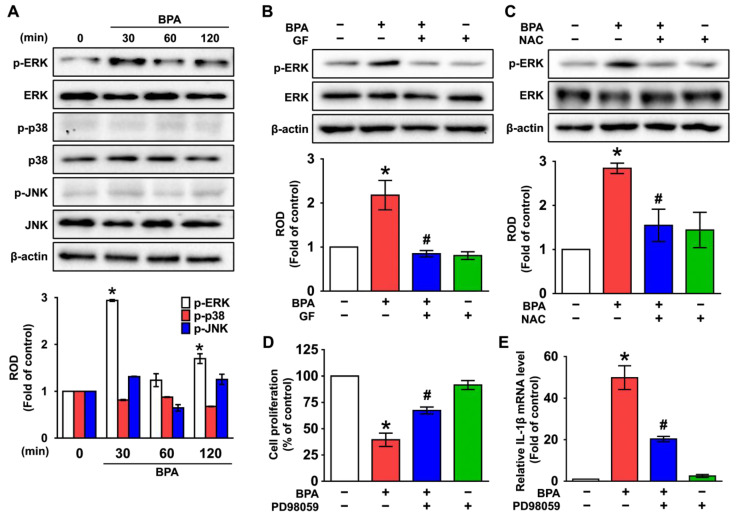
GF inhibits the phosphorylation of ERK in BPA-induced NHDFs. (**A**) Time-dependent response of phosphorylation of MAPKs in NHDFs treated with BPA is shown. n = 4. * *p* ≤ 0.05 vs. 0 min. ROD, relative optical density. (**B**) The inhibitory effect of GF on phosphorylation of ERK in BPA-treated NHDFs is shown. n = 4. * *p* ≤ 0.05 vs. Cont., # *p* ≤ 0.01 vs. BPA alone. (**C**) NHDFs were incubated with NAC for 30 min prior to BPA treatment for 30 min. n = 4. * *p* ≤ 0.05 vs. Cont. # *p* ≤ 0.01 vs. BPA alone. NHDFs were pretreated with ERK inhibitor, PD98059 (1 μM) for 30 min prior to BPA exposure for 6 h. n = 4. The level of cell proliferation (**D**) and IL-1β mRNA (**E**) is shown. * *p* ≤ 0.01 vs. control. # *p* ≤ 0.01 vs. BPA alone. n = 4. The green squares indicate the cells treated with GF, NAC, or PD98059 alone.

The functional role of BPA on the activity of mitogen-activated protein kinases (MAPKs), which are well-known mediators for cell death and inflammation at the downstream factor of ROS [35,36,37,38], was investigated. The activation of ERK was increased at 30 min by treatment with BPA, while p38 MAPK and JNK were not induced by BPA (Figure 4A), and its effect at 30 min was significantly inhibited by treatments with GF (Figure 4B). Interestingly, increased phosphorylation of ERK was blocked by an antioxidant, NAC (Figure 4C), indicating that the phosphorylation of ERK is regulated by the production of ROS. Furthermore, cytotoxicity (Figure 4D) and IL-1β expression (Figure 4E) augmented by BPA were significantly restored by pretreatment with the ERK inhibitor, PD98059. These data indicate that ERK phosphorylation mediated by ROS is a necessary step for inflammatory skin dermal damage and that the signaling cascade triggered by BPA can be significantly inhibited by treatment with GF.

### 3.4. GF Regulates the Activation of NF-κB Responsible for the IL-1β Expression Triggered by BPA

**Figure 5:**
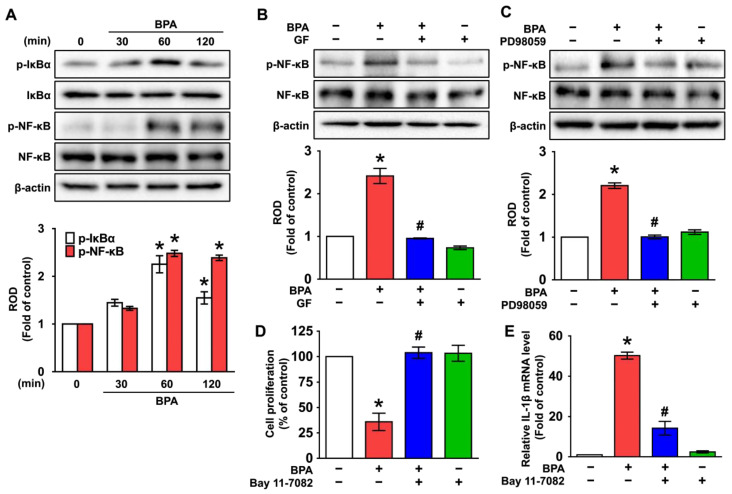
GF inhibits the NF-κB activation to regulate the IL-1β expression triggered by BPA. (**A**) Time-dependent responses of activation of IκBα and NF-κB in NHDFs exposed by BPA are shown. n = 3. * *p* ≤ 0.05 vs. 0 min. ROD, relative optical density. (**B**) The inhibitory effect of GF on activation of NF-κB in BPA-treated NHDFs is shown. n = 4. * *p* ≤ 0.05 vs. Cont., # *p* ≤ 0.05 vs. BPA alone. (**C**) NHDFs were pretreated with PD98059 for 30 min prior to BPA exposure for 60 min. n = 4. * *p* ≤ 0.05 vs. Cont. # *p* ≤ 0.01 vs. BPA alone. NHDFs were pretreated with NF-κB inhibitor, Bay 11-7082 (1 μM), for 30 min prior to BPA exposure for 6 h. The level of cell viability (**D**) and IL-1β mRNA (**E**) is shown. * *p* ≤ 0.05 vs. Cont. # *p* ≤ 0.05 vs. BPA alone. n = 4. The blue squares indicate the cells treated with GF, PD98059, or Bay 11-7082 in the presence of BPA. The green squares indicate the cells treated with GF, PD98059, or Bay 11-7082 alone.

The role of GF in the phosphorylation of the redox-sensitive transcription factor, nuclear factor-kappa B (NF-κB) [39,40], was subsequently examined. BPA significantly increased the phosphorylation of IκBα and NF-κB at 60 and 120 min (Figure 5A). However, NF-κB activation was significantly inhibited by GF and the ERK inhibitor, PD98059 (Figure 5B,C), demonstrating that GF blocks NF-κB phosphorylation mediated by ERK in BPA-treated NHDFs. Interestingly, the increases in dermal cytotoxicity (Figure 5D) and IL-1β expression (Figure 5E) that occurred due to BPA were significantly decreased after incubation with the inhibitor for NF-κB (Bay 11-7082), suggesting that the phosphorylation of NF-κB is involved in inflammatory cytotoxic cell death triggered by BPA. These data indicate that NF-κB activation mediated by ERK plays a critical role in the promotion of cell death and inflammation initiated by BPA and that the BPA signaling pathway can be negatively regulated by GF.

### 3.5. GF Blocks Dermal Fibroblastic Apoptosis and Inflammation Caused by BPA

**Figure 6:**
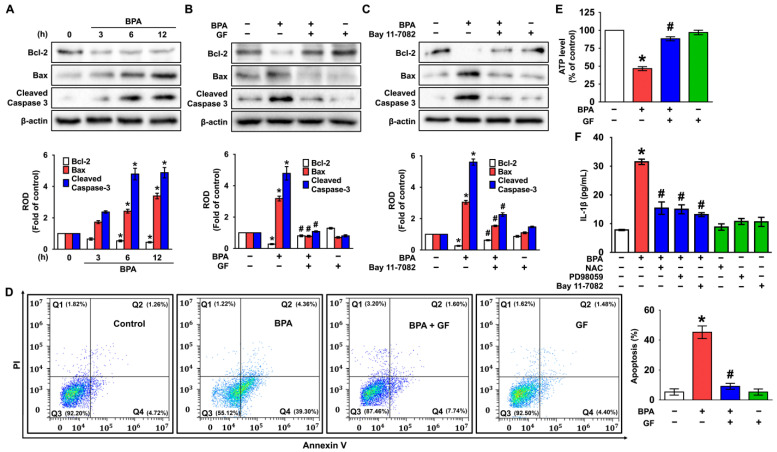
GF blocks dermal fibroblastic apoptosis and inflammation caused by BPA. (**A**) Time-dependent response of expression of cleaved caspase-3, Bcl-2, and Bax in NHDFs exposed by BPA are shown. n = 3. * *p* ≤ 0.05 vs. 0 h. ROD, relative optical density. (**B**) The inhibitory effects of GF on expression of apoptosis-related proteins in BPA-treated NHDFs are shown. n = 4. * *p* ≤ 0.01 vs. Cont., # *p* ≤ 0.01 vs. BPA alone. (**C**) NHDFs were pretreated with Bay 11-7082 for 30 min prior to BPA exposure for 6 h. n = 4. * *p* ≤ 0.01 vs. Cont. # *p* ≤ 0.01 vs. BPA alone. (**D**) NHDFs were incubated with GF for 30 min prior to BPA exposure for 6 h. The apoptotic cell proportion stained by Annexin V/PI was analyzed by performing flow cytometry. n = 4. * *p* ≤ 0.01 vs. control. (**E**) The level of ATP production regulated by GF in BPA-treated NHDF for 6 h. * *p* ≤ 0.05 vs. Cont. # *p* ≤ 0.01 vs. BPA alone. n = 4. (**F**) Cells were treated with NAC, PD98059, and Bay11-7082 for 30 min prior to BPA exposure for 6 h. w is shown. * *p* ≤ 0.01 vs. Cont. # *p* ≤ 0.05 vs. BPA alone. n = 4. The green squares indicate the cells treated with GF, NAC, PD98059, or Bay 11-7082 alone.

Having suggested the crucial role of NF-κB in the regulation of dermal fibroblastic cell death [18,41,42], the correlation of the phosphorylated NF-κB with the apoptosis-related proteins and IL-1β secretion was examined. BPA decreased the expression of Bcl-2, while significantly inducing the expression of cleaved caspase-3 and Bax (Figure 6A). However, the level of apoptosis-related proteins could be regulated by treatment with GF and the NF-κB inhibitor, Bay 11-7082 (Figure 6B,C), indicating that GF inhibits the expression of mitochondrial apoptotic factors mediated by NF-κB in BPA-treated NHDFs. To confirm whether BPA induces apoptotic death in human dermal fibroblasts, flow cytometric analyses were further performed. BPA significantly stimulated the apoptosis of NHDFs, while for necrosis, a marginal effect was noted. However, GF markedly inhibited apoptotic cell death triggered by BPA (Figure 6D). On the other hand, the level of ATP was reduced by BPA treatment, even though the decreases could be blocked by GF (Figure 6E). These results suggest that GF is a functional substance with pharmacological activity, while also having an inhibitory effect on mitochondrial apoptotic cell death accompanied by a reduction of the ATP level in NHDFs exposed to BPA. Moreover, the secretion of IL-1β induced by BPA was significantly attenuated by treatment with the various inhibitors related to the cytotoxic signaling pathway including NAC, PD98059, and Bay11-7082 in NHDFs (Figure 6F). This indicates that BPA regulates IL-1β production involved in the induction of skin dermal fibroblastic inflammation through ROS/ERK/NF-κB pathways.

### 3.6. GF Inhibits Dermal Fibroblastic Pyroptosis Caused by BPA

**Figure 7:**
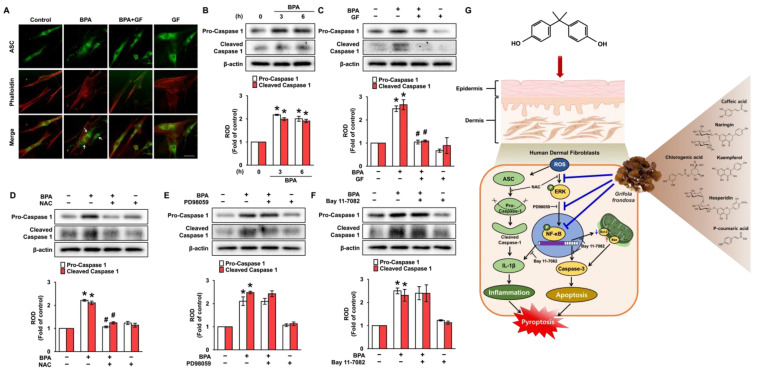
GF inhibits dermal fibroblastic pyroptosis caused by BPA. (**A**) The inhibitory effect of GF on ASC speck formation (green) confirmed by confocal microscopy is shown. Phalloidin was used for F-actin counterstaining (red). Scale bars, 100 μm (magnification × 400). n = 4. (**B**) Time-dependent responses of expression of pro- and cleaved caspase-1 exposed by BPA are shown. n = 4. * *p* ≤ 0.01 vs. 0 h. ROD, relative optical density. (**C**) The inhibitory effects of GF on expression of pro- and cleaved caspase-1 in BPA-treated NHDFs are shown. n = 4. * *p* ≤ 0.01 vs. Cont. # *p* ≤ 0.05 vs. BPA alone. NHDFs were exposed to NAC (**D**), PD98059 (**E**), and Bay 11-7082 (**F**) for 30 min prior to BPA treatment for 6 h. n = 4. * *p* ≤ 0.05 vs. Cont. # *p* ≤ 0.01 vs. BPA alone. (**G**) The sequences of presumed signaling pathways regulated by GF are summarized.

To further understand how BPA regulates IL-1β production, the role of BPA in the speck formation of pyrin domain of the adaptor protein, ASC (apoptosis-associated speck-like protein containing a CARD) [10,11] was checked (Figure 7). The ASC speck formation was presented at 6 h by treatment with BPA, and its pyroptotic effect was significantly inhibited by treatments with GF (Figure 7A). An increase in the pro- and cleaved forms of caspase-1 was detected after 6 h of BPA treatment (Figure 7B). However, the activation of caspase-1 induced by BPA was markedly inhibited by treatments with GF (Figure 7C) as well as NAC (Figure 7D), but not by PD98059 (Figure 7E) or Bay 11-7082 (Figure 7F). These results indicate that ROS production is involved in the caspase-1 activation mediated by inflammasome formation responsible for the secretion of IL-1β protein triggered by BPA, and GF has the ability to block dermal fibroblastic pyroptosis caused by BPA.

## 4. Discussion

In the present study, the overall findings provide important evidence that bisphenol A (BPA) triggers the signaling cascade of pyroptotic skin dermal damage and that *Grifola frondosa* (GF) containing naringin, hesperidin, chlorogenic acid, and kaempferol inhibits apoptotic and inflammatory pathways triggered by BPA through blocking the ROS/ERK/NF-κB axis (Figure 7G). Regarding the linkage of BPA to an elevated risk of health issues, it should be noted that the toxicological mechanism of BPA exposure by ingestion of water and food is relatively well established. However, the impact of BPA on dermal exposure and skin absorption is not well documented, despite the 46% of BPA that is permeated across the skin [4]. Indeed, studies concerning the pathophysiological role of BPA during cutaneous absorption have been much less published when compared to those related to the respiratory [43], reproductive [44] and urinary systems [45]. In the present study, BPA was found to stimulate cytotoxicity and IL-1β expression in promoting skin dermal damage. These results indicate that the potential pathogenesis of BPA via dermal contact is related to excessive or uncontrolled levels of apoptosis and inflammation leading to skin damage, which is the most common consequence of the cumulative changes in skin structure, function, and appearance. This is the first study to show the mechanism by which BPA induces the speck formation of ASC and the caspase-1-mediated IL-1β production involved in the induction of skin dermal fibroblastic inflammation and apoptosis through ROS/ERK/NF-κB pathways, thus demonstrating the relevant nature of BPA in skin toxicology and physiology.

Given that BPA is one of the well-known endocrine-disrupting chemicals (EDC) that causes inflammatory and allergic skin diseases, chloracne, disorders of skin pigmentation, skin cancer, and skin aging [10], it is crucial to develop the functional substances that control the mechanism of action as induced by BPA [45]. Recently, various bioactive and pharmacological properties of GF have been considered in ameliorating cancer, inflammation, hyperlipemia, hypertension, and diabetes [46,47]. Despite the ample evidence showing that GF has a variety of pharmacological applications, the anti-pyroptotic mechanism of GF to block the oxidative BPA signaling pathway in skin dermal fibroblasts has not been documented yet. The present study suggests several interesting aspects considering the functional role of GF. First, GF containing high amounts of naringin and hesperidin has the ability to inhibit the pyroptotic signaling pathway of skin dermal fibroblasts by scavenging ROS production in NHDFs treated with BPA. Many studies have focused on the constituents of GF extracts with different extraction solvents [48,49,50,51]. Although the constituents of GF are similar to the previous study [48], the results of the UPLC analysis indicate that the amounts of naringin and hesperidin in GF water extract were much higher than in the previous study. The discrepancy with regard to the amount of bioactive compounds presented in GF extracts may be due to differences in the extraction conditions, where GF was soaked in ddH_2_O and incubated at 25 °C for 72 h. In addition, naringin and hesperidin are flavonoids found in the plants of citrus fruits and have been reported to show various pharmacological benefits such as antioxidant, antimicrobial, anti-inflammatory, antiapoptotic, and antimutagenic activities [52,53]. Moreover, many reports have insisted that bioactive compounds such as phenolic acids (caffeic acid, chlorogenic acid and p-coumaric acid) and flavonoids (kaempferol, naringin, and hesperidin) as shown in the UPLC analysis of GF are critical phytochemical elements that attract considerable attention both in terms of healthy nutrition and production of antioxidant foods [52,53,54,55,56]. On the other hand, BPA has been shown to amplify oxidative stress by increasing the ROS accumulation responsible for necroptosis, apoptosis, ferroptosis, autophagy and pyroptosis [32,34]. These results are consistent with previous findings revealing that the oxidative cytotoxicity initiated by ecotoxicological BPA can be suppressed by treatment with phenolic and flavonoid compounds isolated from various plants and fungi [51,57]. This evidence also indicates that polyphenol/flavonoid-rich GF is a potential therapeutic antioxidant that can attenuate oxidative stress-related diseases that affect inflammatory skin dermal damage exposed by BPA.

Second, the present findings are the first to show that GF acting through ROS has the ability to block apoptotic cell death and IL-1β production via regulation of ERK/NF-κB activity induced by BPA. Earlier works have indicated that ROS is directly associated with the regulation of MAPK including JNK, p38 MAPK, and ERK1/2 to govern the expression of a variety of cellular proteins and transcription factors responsible for oxidative skin dermal damage in response to an external stimulus [39,40]. In addition, the present study noted that ERK activation, as uniquely stimulated by ROS production, is required for apoptotic cell death and IL-1β production induced by BPA, suggesting that BPA selectively regulates specific MPAK isozymes governed by the oxidative signaling pathway in promoting inflammatory skin dermal damage. On the other hand, these relevant data are further supported by a previous study showing that GF uniquely inhibits the phosphorylation of ERK stimulated by ROS in vascular endothelial growth factor (VEGF)-treated umbilical vein endothelial cells [58]. Thus, the present findings strongly suggest that the therapeutic effect of GF on the aberrant activation of dermal ERK is carried by its antioxidant capacity against BPA. Having demonstrated that ROS and ERK are major effector molecules of GF in BPA-treated NHDFs, the current results further suggest that GF negatively controls the activation of NF-κB mediated by ERK, which is required for the apoptotic pathway triggered by BPA during the promotion of skin dermal damage. The phosphorylation of NF-κB is an important transcriptional mechanism during the promotion of oxidative tissue damage and inflammation induced by BPA [59]. Earlier reports have proven that NF-κB phosphorylated by external/internal stimuli such as ROS and MAPK is segregated from IκBα to translocate into the nucleus where it has a critical role as one of the multifaceted transcriptional factors in the regulation of apoptotic gene expression [18,41,42]. Indeed, it was reported that phosphorylated cytoplasmic ERK1/2 has the ability to translocate to the nucleus to activate a variety of transcriptional factors including NF-κB [60]. Thus, it is conceivable that GF has the potential to abrogate the oxidative phosphorylation of NF-κB by suppressing the ERK pathway in BPA-treated NHDFs. Many studies have reported that the ERK–NF-κB axis plays a critical role in the cell proliferation/survival pathway of various malignant tumors such as osteosarcoma [61], breast cancer [62], pancreatic cancer [63], oral cancer [64] and hepatocellular carcinoma [65]. Unlike the pro-tumorigenic roles, the ERK–NF-κB axis is also involved in the apoptosis pathway of hepatic ischemia reperfusion [66], renal injury [67], reproductive toxicity in epididymis [68], and skin dermal injury [36]. These controversial issues of the ERK–NF-κB axis come from the presence of the different receptors, stimuli, and proteins in different cell types. Thus, the current data strongly suggest that the physiological meanings of the ERK–NF-κB axis induced by other carcinogens are different from the present results revealing that the BPA signaling pathway regulates skin dermal fibroblastic apoptosis. Together, the overall findings indicate that the activation of ERK and NF-κB is a predominant mechanism in the promotion of apoptosis and inflammation responsible for skin dermal damage initiated by BPA.

Finally, GF was found to have the ability to regulate the transcriptional expression of apoptosis proteins (Bcl-2, Bax, and cleaved caspase-3) via the blocking of NF-κB activity in NHDFs treated with BPA. As the key components of apoptosis, Bcl-2 and Bax have been shown to play an important role in the delivery of death signals into mitochondria sites that trigger the catastrophic transformation of mitochondrial function [69]. Having shown that BPA provokes oxidative stress and thus induces the apoptotic signaling pathway mediated by the mitochondria via Bax oligomerization [70], it was proven that mitochondrial translocation of the Bax protein is accompanied by a significant increase in caspase-9 activation and cytochrome c release responsible for caspase-3 activation. On the other hand, Bcl-2 has been reported to play an important role in cell survival and in attenuating apoptotic gene expression in the mitochondrial outer membrane. Moreover, the present findings have proven that the blocking of NF-κB in NHDFs normalizes the levels of Bcl-2, Bax, and cleaved caspase-3, suggesting the transcriptional activity of NF-κB influences the occurrence of apoptotic cell death elicited by BPA. This evidence is supported by a previous study showing that phosphorylated NF-κB significantly regulates the expression of Bax and Bcl-2 by binding to their promoter regions [18]. Importantly, the present results indicate that GF significantly restores the levels of ATP affected by BPA. Given that BPA ingestion reduces the activity of mitochondrial respiratory chain complexes in the liver [71], the current findings indicate that BPA stimulates the mitochondrial dysfunction related to oxidative stress and apoptosis in skin dermal fibroblasts. Importantly, BPA has the ability to induce the caspase-1 activation mediated by ROS production to induce the IL-1β maturation, whereas GF showed a marginal effect and appeared to normalize the inflammatory response induced by BPA. Given that inflammasome is a cytoplasmic multiprotein oligomer that initiates an inflammatory form of cell death related to the releasing mature pro-inflammatory cytokine IL-1β [72,73], the present data indicate that BPA triggers the caspase-1 activation controlled by the binding of NLR proteins and ASC adaptors in the secretion of IL-1β, and, intriguingly, that the caspase-1 activation is strongly dependent on ROS production associated with the apoptotic signaling pathway. Thus, it is possible that apoptotic cell death induced by BPA coupled with the production of IL-1β is crucial role in the inflammatory skin dermal fibroblastic damage.

The current findings are the first to demonstrate the signaling cascade of inflammatory apoptotic cell death associated with IL-1β production in skin dermal fibroblasts during BPA exposure. Collectively, the pharmacological activities of GF represented by the present results strongly indicate that GF is a potent candidate as a therapeutic skin agent to inhibit the pyroptotic signal pathway regulated by oxidative stress responsible for inflammatory dermal fibroblastic skin damage induced by BPA.

## 5. Conclusions

The overall findings from current data indicate that the pharmacological effect of GF in ameliorating the oxidative BPA signaling pathway is responsible for dermal fibroblastic skin inflammation and apoptotic cell death, which potentially leads to skin wrinkles, aging, and inflammatory diseases. Moreover, the present results define the relevant mechanism of the polyphenol and flavonoid-rich GF for blocking the ERK/NF-κB signaling cascade and the caspase-1 activation, which are all associated with ROS production during BPA treatment and could be crucial in the development of pharmacological and cosmetic agents against cutaneous dermal damage. Further research remains to be done on the role of GF against the oxidative BPA signaling pathway in an in vitro artificial human skin equivalent model that is a three-dimensionally cultured skin model to mimic the morphology and physiology of the human skin dermis.

## Figures and Tables

**Table 1:** The analysis condition of kaempferol, hesperidin, naringin, p-coumaric acid, caffeic acid, and chlorogenic acid. FA—formic acid.

Time (min)	0.1% FA/Water (%)	0.1% FA/Acetonitrile (%)
0	98	2
1.5	98	2
2.0	90	10
4.0	70	30
6.0	70	30
7.0	60	40
9.0	30	70
10.0	5	95
14.0	98	2
16.0	98	2

**Table 2:** PCR primer sequences.

Gene	Identification	Primer Sequence, 5′-3′
IL-1β	Forward	TTCGAGGCACAAGGCACAAC
	Reverse	GTGGTGGTCGGAGATTCGTA
TNF-α	Forward	CTCCTCACCCACACCATCA
	Reverse	GGAAGACCCCTCCCAGATAG
IL-6	Forward	CAATAACCACCCCTGACCCAA
	Reverse	ACCAGAAGAAGGAATGCCCA
β-actin	Forward	AACCGCGAGAAGATGACCCAGATCATGTTT
	Reverse	AGCAGCCGTGGCCATCTCTTGCTCGAAGTC

**Table 3:** Total polyphenol and flavonoid contents of GF extract.

Total Polyphenol	Total Flavonoid
53.30 μg/mL	59.28 μg/mL

**Table 4:** The constituents of GF analyzed by UPLC.

Compound	Area (mV × s)	Height (mm)	Content (mg/L)
Naringin	31,172	3025	18.183 ± 0.962
Hesperidin	17,915	3335	7.488 ± 0.270
P-coumaric acid	5679	1763	4.794 ± 0.176
Chlorogenic acid	16,961	5422	4.688 ± 0.440
Kaempferol	24,594	5920	4.169 ± 0.227
Caffeic acid	5690	1720	1.887 ± 0.148

## Data Availability

Data available on request due to restrictions, e.g., privacy or ethical. The data presented in this study are available on request from the corresponding author.
